# Global Epidemiology of Buruli Ulcer, 2010–2017, and Analysis of 2014 WHO Programmatic Targets

**DOI:** 10.3201/eid2512.190427

**Published:** 2019-12

**Authors:** Till F. Omansen, Alfred Erbowor-Becksen, Rie Yotsu, Tjip S. van der Werf, Alexander Tiendrebeogo, Lise Grout, Kingsley Asiedu

**Affiliations:** Bernhard Nocht Institute for Tropical Medicine and University Medical Center Hamburg-Eppendorf, Hamburg, Germany (T.F. Omansen);; World Health Organization, Geneva, Switzerland (T.F. Omansen, A. Erbowor-Becksen, L. Grout, K. Asiedu);; Indiana University, Indianapolis, Indiana, USA (A. Erbowor-Becksen);; Nagasaki University, Nagasaki, Japan (R. Yotsu);; National Center for Global Health and Medicine, Tokyo, Japan (R. Yotsu);; University of Groningen, Groningen, the Netherlands (T.S. van der Werf);; World Health Organization Regional Office for Africa, Brazzaville, Republic of the Congo (A. Tiendrebeogo)

**Keywords:** Buruli ulcer, *Mycobacterium ulcerans*, epidemiology, programmatic targets, World Health Organization, tuberculosis and other mycobacteria, bacteria, Australia, Democratic Republic of the Congo, DRC, Nigeria, Gabon, Papua New Guinea, Japan, Benin, Cameroon, Côte d’Ivoire, Ghana, Guinea, Liberia, Togo

## Abstract

Buruli ulcer is a neglected tropical disease caused by *Myocobacterium ulcerans*; it manifests as a skin lesion, nodule, or ulcer that can be extensive and disabling. To assess the global burden and the progress on disease control, we analyzed epidemiologic data reported by countries to the World Health Organization during 2010–2017. During this period, 23,206 cases of Buruli ulcer were reported. Globally, cases declined to 2,217 in 2017, but local epidemics seem to arise, such as in Australia and Liberia. In 2013, the World Health Organization formulated 4 programmatic targets for Buruli ulcer that addressed PCR confirmation, occurrence of category III (extensive) lesions and ulcerative lesions, and movement limitation caused by the disease. In 2014, only the movement limitation goal was met, and in 2019, none are met, on a global average. Our findings support discussion on future Buruli ulcer policy and post-2020 programmatic targets.

*Mycobacterium ulcerans* causes the neglected tropical skin disease Buruli ulcer (*1*). The infection manifests as a nonulcerative nodule, plaque, or edema, which ulcerates within 4–6 weeks and develops the characteristic undermined edges and yellowish-white necrotic slough (Figure 1) (*2*). The disease is diagnosed by its characteristic clinical features and confirmed in the laboratory using histopathology, microbiological culture, and PCR for the IS*2404* mycobacterial insertion sequence element (*3*). There is no efficient vaccine for Buruli ulcer (*4*), and disease control strategy focuses on early case detection and comprehensive treatment of individual patients. Treatment of Buruli ulcer has experienced a paradigm shift during the past 2 decades, from surgery to an 8-week course of the antimicrobial drugs rifampin and clarithromycin (*5*,*6*). Recent preclinical animal experiments suggest that a higher dose of rifampin can dramatically increase efficacy and reduce treatment duration (*7*–*9*).

**Figure 1:**
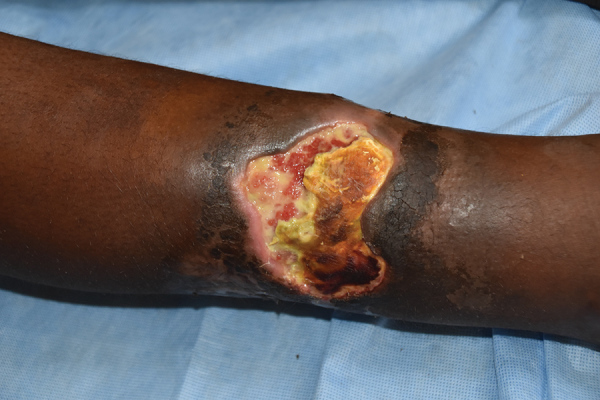
Typical Buruli ulcer lesion on the arm of a patient from Ghana. Central necrosis, yellowish-white slough, and undermined edges surround the wound. Photo courtesy of T.S. van der Werf.

*M. ulcerans* is an environmental pathogen often associated with aquatic environments. The DNA of the organism has been found in aquatic insects (*10*), mosquitoes (*11*), and domestic animals (*12*). Experimental puncturing injury resulting in introduction of organisms into mouse skin and subcutis led to infection (*13*). However, transmission pathways in nature are complex and multifactorial and depend on the local ecosystem. 

A definitive transmission pathway of *M. ulcerans* has not been described. *M. ulcerans* was first described as the causative agent of Buruli ulcer in Victoria, Australia, in 1948 (*14*), while descriptions of ulcerative lesions probably caused by *M. ulcerans* in Africa, namely Uganda, date back to the late 18th century. Formal description and reporting of cases on the continent of Africa occurred during the 1950s and 1960s (*15*). Buruli ulcer has been reported in 33 countries worldwide, occurring mainly in West Africa and southeastern Australia (*1*). The disease occurs in very concentrated, small geographic foci within countries, as described in Cameroon and Australia (*16*,*17*). Increases in cases have been associated with heavy periods of rainfall in some places (*18*–*21*). In Africa, landscape fragmentation and destruction has been suggested as a risk factor for Buruli ulcer (*22*). 

The niche, ecology, and transmission of the environmental human pathogen *M. ulcerans* are, in summary, poorly understood; close epidemiologic surveillance is important for disease control, and drivers of local occurrence of the disease should be closely investigated. A shift of the endemic focus has been described in Australia (*23*). Because the exact transmission route remains unknown, no clear recommendations can be given on Buruli ulcer prevention. The main strategy for Buruli ulcer disease control is early detection and administration of efficient treatment.

The first global recognition and move toward Buruli ulcer advocacy and research was held in Yamoussoukro, Côte d’Ivoire, in 1998 and resulted in the Yamoussoukro Declaration on Buruli ulcer (*24*). The meeting leaders stressed the importance of the rising burden of Buruli ulcer cases, particularly in West Africa, and called policy makers to action to support the control of the disease. In 2004, the World Health Assembly (WHA) adopted resolution WHA 57.1, calling for enhanced surveillance and control of the disease (https://www.who.int/neglected_diseases/mediacentre/WHA_57.1_Eng.pdf). In 2009, a second high-level meeting held in Benin resulted in the Cotonou Declaration on Buruli ulcer (*25*), calling for greater political commitment for control through early detection and antimicrobial treatment, as well as support for research. At the 2013 World Health Organization (WHO) meeting on Buruli ulcer control and research in Geneva, Switzerland, participants defined 4 programmatic targets to be met by disease-endemic countries by the end of 2014. The targets addressed PCR confirmation, lesion size, and ulceration as indicators of disease progression or severity (late reporting), as well as functional limitation as a reflection of disability. We discuss the current epidemiology of Buruli ulcer and present an analysis, based on data reported to WHO, on progress toward these programmatic targets.

## Materials and Methods

### Data Collection

Buruli ulcer is diagnosed clinically in most settings in which it is endemic; where possible, cases are confirmed by PCR targeting the insertion sequence 2404 (IS*2404*). In addition, microscopy, histopathology, and microbiological culture are used to aid in the diagnosis of Buruli ulcer. A suspected Buruli ulcer case is defined as a clinically diagnosed case. Individual data collected for each suspected BU case are standardized throughout the disease-endemic countries and include demographic characteristics, clinical history, referral, clinical presentation, lesion size category, laboratory confirmation (if available), treatment and dosages, and treatment outcome. Lesions are categorized by diameter to reflect severity: category I, <5 cm; category II, 5–15 cm; and category III, >15 cm diameter or presence of multiple lesions at critical anatomic locations affected (e.g., eye, genitalia). Staff record patient data on the paper-based BU01 form (https://www.who.int/buruli/control/ENG_BU_01_N.pdf) and then summarize the data into a BU register, the BU02 form (https://www.who.int/buruli/control/BU02%20form.pdf). The health facility forwards BU02 forms to district public health officers, who enter the data into a digital spreadsheet submitted to the national BU control program. At the national level, all data are compiled, cleaned, aggregated, and analyzed. 

Buruli ulcer–endemic countries reported data to WHO annually to assess programmatic indicators. The 4 programmatic targets set in 2013 were as follows: 1) >70% of cases reported from any district or country should have been confirmed by a positive PCR; 2) by the end of 2014, the proportion of category III lesions reported from any district or country should have been reduced from the 2012 average of 33% to <25%; 3) by the end of 2014, the proportion of ulcerative lesions at diagnosis reported from any district or country should have been reduced from the 2012 average of 84% to a maximum of 60%; 4) by the end of 2014, the proportion of patients with limitations of movement at diagnosis reported from any district or country should have been reduced from the 2012 average of 25% to a maximum of 15% (*26*). Countries also reported total number of cases, gender distribution, the proportion of patients <15 years of age, the percentage of cases that are located on the lower limb, and the percentage of patients who completed antimicrobial therapy.

These data concerning the programmatic indicators were retrospectively entered into the WHO integrated data platform (WIDP). The WIDP is a web-based open source platform, District Health Information System 2 (DHIS2) (*27*). WHO further adapted WIDP to streamline global reporting from member states to WHO, integrate data from different sources, and strengthen data collection, analysis, and use in disease-endemic countries.

### Data Analysis

We included data reported to WHO during 2010–2017 in this descriptive analysis. We reviewed data from all 33 countries that had ever reported Buruli ulcer, using case numbers, the proportion of patients <15 years of age, sex distribution, lesion location on the lower limb, and antimicrobial treatment completion as descriptive statistics. We calculated incidence rates for Buruli ulcer on the basis of United Nations median population estimates for 2017 (http://data.un.org). Programmatic target indicators are shown per year per country, as available (Table 2); we calculated the global average from country means, which were weighted by their population. We performed statistical analysis and graphing using GraphPad Prism version 7.0a (https://www.graphpad.com), quantumGIS version 2.18.13 (https://www.qgis.org), and RStudio version 1.1.456 (https://rstudio.com).

**Table 2:** Epidemiologic data on Buruli ulcer cases reported to the World Health Organization, 2010–2017*

Region and country			Total no. cases, 2011–2017	2017 data				
	No. suspected cases			Incidence, cases/100,000 population	Patients age <15 y, %	Female patients, %	Lesion located on lower limb, %	Completed antimicrobial therapy, %
	2010	2017						
AFRO region								
Benin	572	267	3,027	2.35	41	50.5	61†	100†
Cameroon	287	No data	1,180	No data	31†	49†	74†	99†
Congo	107	No data	207	No data	No data	No data	No data	No data
Côte d'Ivoire	2,533	344	8,713	1.31	48	52	57†	100†
DRC	136	91	1,535	1.80	33†	44†	72†	100†
Gabon	65	45	402	2.12	40	49	77†	84
Ghana	1,048	538	4,828	1.91	13	48	83†	No data
Guinea	24	98	549	0.83	14†	No data	No data	No data
Liberia	No data	219	353	4.55	14	47	No data	57
Nigeria	7	259	747	0.13	50	57	78†	94
Sierra Leone	No data	No data	28	No data	No data	No data	No data	No data
South Sudan	4	No data	4	No data	No data	No data	No data	No data
Togo	67	62	500	0.76	53	42	54†	86†
AFRO subtotal‡	4,850	1,923	22,073		31	50	71	70
WPRO region								
Australia	42	283	1,033	1.21	10	48	58	100†
Japan	9	6	52	0.0048	17	67	50†	100
Papua New Guinea	5	5	48	0.07	80	60		
WPRO subtotal‡	56	294	1,133		11	49	58	100
Global total	4,906	2,217	23,196		26	50	69	74

*Data from Buruli ulcer–endemic countries that reported continuous data for most of the years assessed. Up-to-date country data on annual reported cases are available at http://apps.who.int/gho/data/node.main.A1631. AFRO, WHO African Region; DRC, Democratic Republic of the Congo; WPRO, WHO Western Pacific Region; WHO, World Health Organization.  †2016 data; 2017 data were not available. ‡Cases and total cases represent sums of countries per region. Programmatic indicators represented mean proportions weighted for case burden in the respective countries.

## Results

### Reporting and Completeness

We analyzed available data from a total of 16 countries: Australia, Democratic Republic of the Congo (DRC), Nigeria, Gabon, Papua New Guinea, Japan, Benin, Cameroon, Côte d’Ivoire, Ghana, Guinea, Liberia, Sierra Leone, South Sudan, Republic of the Congo, and Togo. We excluded Republic of the Congo, Sierra Leone, and South Sudan from the programmatic target analysis because they provided insufficient data; we excluded Burkina Faso, Central African Republic, Sri Lanka, Brazil, Malaysia, China, Angola, Indonesia, Kenya, Malawi, Peru, Senegal, Suriname, Uganda, and Mexico from the analysis because they had not reported relevant data for the study period. 

### Decline of Global Buruli Ulcer Cases and Rise of Local Epidemics 

**Figure 2:**
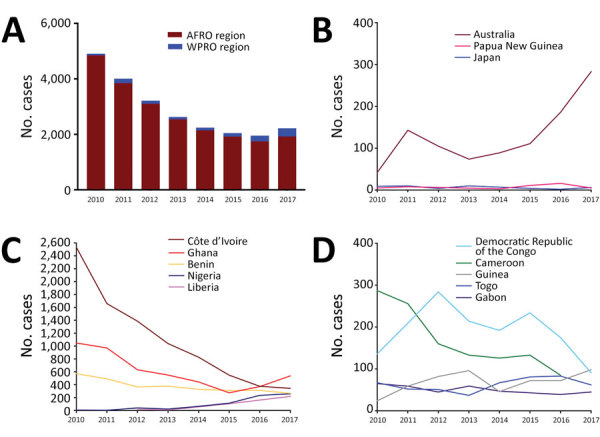
Dynamics of Buruli ulcer epidemiology by cases reported to the World Health Organization (WHO) in 2010–2017. A) Globally, reported cases declined over time, but the proportion of cases reported from WPRO increased. B) WPRO data show an increase in cases in Australia. C) In AFRO, cases drastically declined in Côte d’Ivoire but recently increased in other countries such as Ghana, Nigeria, and Liberia. D) Countries in AFRO that reported fewer cases overall showed stagnant or varying numbers. AFRO, WHO African Region; WPRO, WHO Western Pacific Region.

During 2010–2017, a total of 23,206 cases of Buruli ulcer were reported to WHO by 16 different countries, 14 in the African Region (AFRO) and 3 in the Western Pacific Region (WPRO). In 2017 alone, 2,217 cases of Buruli ulcer were reported globally, 1,923 in AFRO and 294 in WPRO. Overall, the yearly case burden declined from a maximum of 4,906 cases in 2010 to 1,952 cases in 2016; in 2017, however, the number of cases increased to 2,217 cases (Table 1; Figure 2, panel A), mainly driven by a sharp rise in Australia to 283 cases in 2017. Other than Australia, few cases have been reported in WPRO, from Papua New Guinea and Japan (Table 1; Figure 2, panel B); most cases were reported from AFRO. 

**Table 1:** Overview of status on WHO 2014 programmatic targets for Buruli ulcer*

WHO programmatic targets	2012 data	Target set in 2013	2014 data	2017 data
1. PCR confirmation	50%	≥70%	64%	58%
2. Category III lesions	33%	<25%	37%	31%
3. Ulcerative lesions	84%	≤60%	64%	75%
4. Movement limitation	25%	≤15%	15%	17%

*Targets were formulated at the 2013 WHO Buruli Ulcer Research and Control Meeting (*26*). Targets were based on the average of data reported from countries in 2012. They were set to be achieved by the end of 2014. Values represent means weighted for case burden of every country, computed from data reported to WHO. For some countries, information on a certain indicator was not available, if this was the case, the case burden was exempted from the calculation for this specific indicator. Red shading indicates failure to meet target; green shading indicates that the target was met. WHO, World Health Organization.

**Figure 3:**
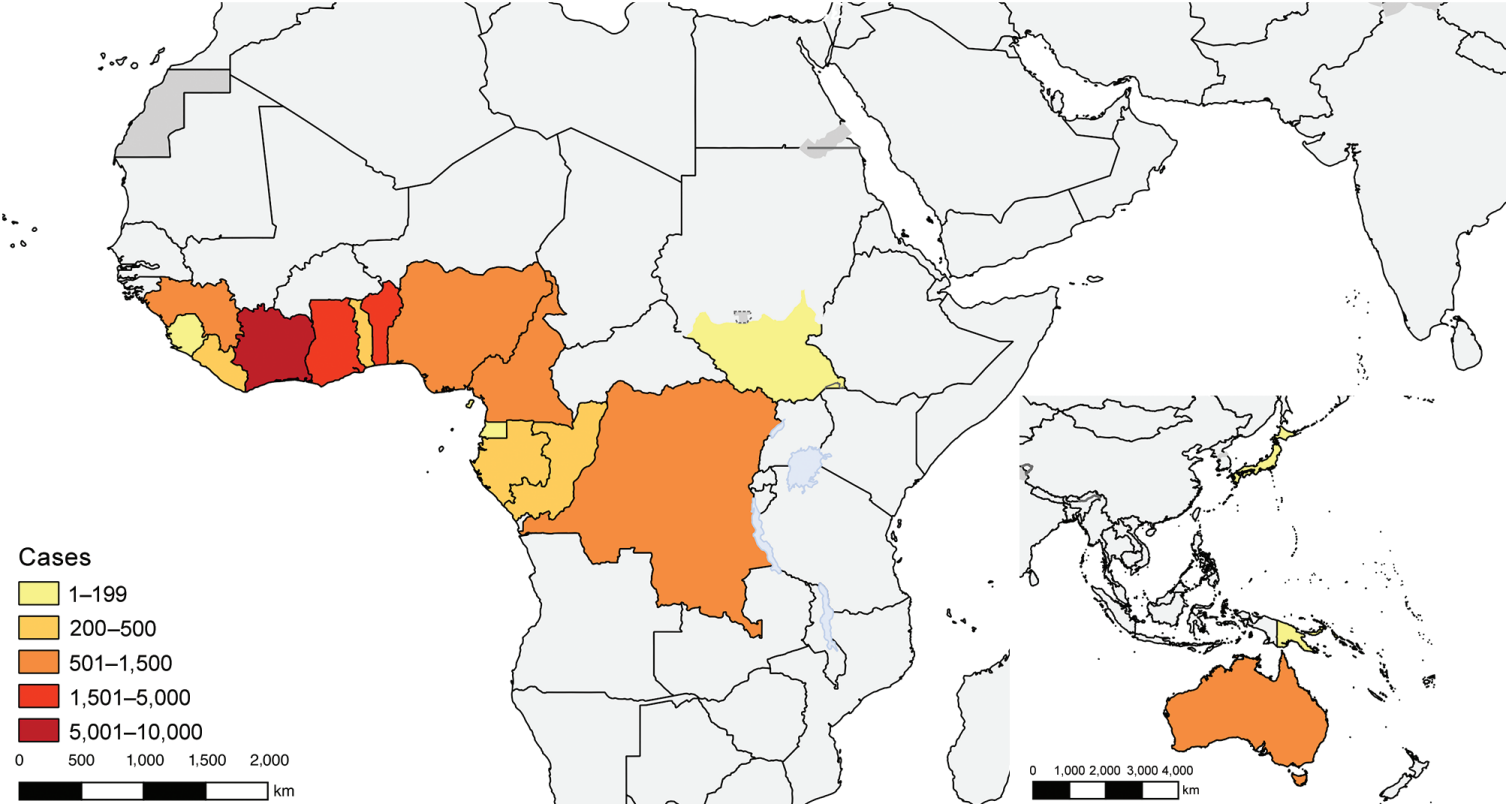
Geographic distribution of Buruli ulcer cases officially reported to World Health Organization during 2010–2017. Concentrations in West Africa and Australia are clearly visible.

Countries reporting >200 cases in 2017 (termed high burden; Figures 2, 3) in Africa were Côte d’Ivoire, Ghana, Benin, Nigeria, and Liberia; within these countries, case numbers have increased in Ghana, Nigeria, and Liberia. Cases were constant in Benin; Côte d’Ivoire saw a decline in cases from a historically high-burden country in 2010 (Figure 2, panel C). Case numbers reported from the remaining low-burden countries, DRC, Cameroon, Guinea, Togo, and Gabon, fluctuate around 20–200 cases/year (Figure 2, panel D). We observed the highest incidences in Liberia (4.55 cases/100,000 population), Benin (2.35 cases/100,000 population), Gabon (2.12 cases/100,000 population), Ghana (1.91 cases/100,000 population), and DRC (1.80 cases/100,000 population) (Table 1).

### Patient Age and Sex

Age information was available for 18,449 of the 23,206 reported Buruli ulcer cases from 2010–2017. Of these cases, 40% occurred in patients <15 years of age. Countries with >40% of cases occurring in children <15 years of age in 2016–2017 were Benin, Côte d’Ivoire, Gabon, Nigeria, and Togo. Countries with <15% of cases occurring in patients <15 years of age were Liberia, Guinea, Ghana, and Australia. Distribution by sex was even globally, with 50% of reported cases occurring in female and 50% in male case-patients.

### Lesion Location

On average, 69% of Buruli ulcer lesions were located on a lower limb. For DRC, Cameroon, Gabon, Nigeria, and Ghana, 70% of recorded lesions were on a lower limb. The lowest values were reported from Japan (50%), Togo (54%), Côte d’Ivoire (57%), and Australia (58%).

### Completion of Antimicrobial Treatment 

Most countries that reported data stated that 99%–100% of patients completed antimicrobial treatment in 2016 and 2017. Togo (86%) and Gabon (84%) reported slightly lower rates of patients who completed the regimen. Low levels of completed antimicrobial treatment were reported from Liberia (57%) and Ghana (22%); these low rates may be due to incomplete or inadequate reporting.

### Progress toward 2014 WHO Targets 

**Figure 4:**
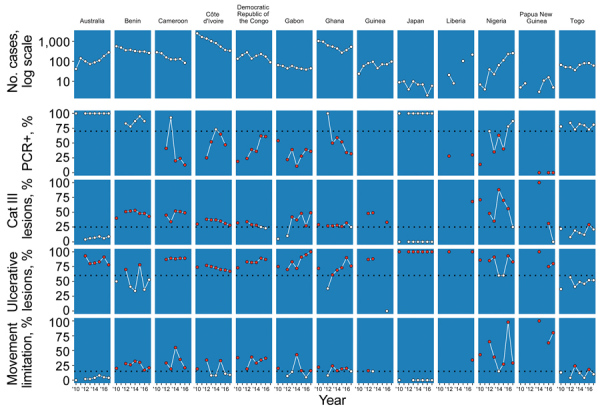
Depiction of progress toward World Health Organization programmatic targets for Buruli ulcer–endemic countries that reported continuous data. Black dotted lines indicate 2014 targets. White dots indicate that the country met the target; red dots indicate that it did not. Cat, category; +, positive.

We used data from 2012 as a baseline measure to formulate the programmatic targets. The global average rate of PCR confirmation in 2012 was 50%. Category III lesions were present in 33% of case-patients, ulcerative lesions in 84%, and movement limitations in 25% (Table 2; Figure 4). By 2014, the rate of confirmation by PCR increased globally to 64%, which did not meet the target of >70%. The number of category III lesions actually increased to 37%, but ulcerative lesions declined to 64%. The only target met by 2014 was target 4, movement limitations, which were reduced to 15%. Subsequently, in 2017, 58% of Buruli ulcer cases were PCR confirmed, 31% of lesions were category III, 75% of lesions were ulcerative, and 17% of patients had movement limitations, as reported by countries (Table 2). Five countries met the PCR confirmation target, 2 countries met the category III target, 3 countries met the ulcerative lesion target, and 5 countries met the movement limitation target (Figure 4).

We observed many differences at the country level. In general, WPRO countries, particularly Australia and Japan, have had very high rates of PCR confirmation and low rates of category III lesions and movement limitation. In the AFRO countries, PCR confirmation was high in Benin and Togo; we observed improved PCR confirmation rates in DRC and Nigeria. PCR confirmation was low in Cameroon and Gabon and had declined in Ghana from 2012 to 2017. In Côte d’Ivoire, the PCR confirmation rate improved to meet the target in 2014 but then declined again by 2017. Category III lesions were low in Togo and recently also in DRC, meeting the targets in most recent years. Benin, Cameroon, and Nigeria in particular had high rates of category III lesions. Ulcerative lesions were common in all countries in both the WPRO and AFRO regions, with the exception of Togo. Ghana, Togo, and Papua New Guinea had low rates of movement limitation, whereas Nigeria, Cameroon, and Benin’s rates of movement limitation exceeded the set target.

We have analyzed data reported through the end of 2018. Figures on the programmatic targets are available on the WIPD web portal (http://extranet.who.int/ntdportal).

## Discussion

Even though overall Buruli ulcer cases declined from 2010 until 2017, some countries such as Nigeria, Liberia, and Australia recently reported an increase in cases. The greatest challenge in Buruli ulcer epidemiology and control is that the reservoir and transmission of *M. ulcerans* are unknown. Reporting bias, differences in reporting, or differences in incidence could cause fluctuation in recorded case burden across regions. Nigeria has recently implemented a national Buruli ulcer program; previously, some Buruli ulcer patients had been treated in neighboring Benin (*28*,*29*). The installation of a formal Buruli ulcer control program and the concurrent intensification of disease control efforts, such as early case finding, might have contributed to increasing reported cases. However, interviewees in a study reported poor knowledge about Buruli ulcer within the local community in one of the affected states of Nigeria, stressing the necessity to further strengthen awareness and control efforts to detect cases (*30*). The number of cases also rose recently in Liberia, the country with the highest incidence of Buruli ulcer (4.55 cases/100,000 population). Underreporting had previously been suggested to be associated with civil war and a lack in knowledge of the disease among healthcare workers (*31*). 

In countries such as Benin, Côte d’Ivoire, and Ghana that have well-established facilities for detection and treatment of Buruli ulcer, changes in epidemiology may be due to environmental drivers that are not yet understood, in addition to probable reporting bias. In addition, some countries, such as Uganda, had been endemic for Buruli ulcer but no longer report it, perhaps because of environmental or population changes. In Australia, Buruli ulcer has been known since the 1930s and is a notifiable disease in the state of Victoria; not only an increase in cases but also an increase in severity of the disease have been reported, and the increases may be attributable to a genomic change in *M. ulcerans* (*32*). *M. ulcerans* is a genetically highly clonal organism, and certain genotypes are confined to 1 geographic region (*33**,**34*). An increase in pathogenicity may be attributed to a genetic shift within the predominant genotype. Changes in the structure of mycolactone or the amount produced could also be driving increased virulence of *M. ulcerans.*

In 2013, WHO formulated programmatic targets to be reached by the end of 2014. The 2014 programmatic targets were defined to ensure good diagnosis (PCR confirmation) and early case finding (fewer category III, ulcerated lesions, movement limitation). Some progress that had been initially achieved toward the programmatic targets was lost soon after, and the situation actually deteriorated below the 2012 average. The overall low rate of 58% of PCR-confirmed infections indicates a need for implementing high-quality PCR locally and training health staff in sample collection, processing, and testing. Of note, PCR diagnosis is universally available in affluent countries, such as Australia. PCR positivity for *M. ulcerans* is part of the case definition in Australia; hence, a rate of 100% PCR confirmation is reported, as expected. In other countries, physicians need to rely on clinical diagnosis or other tests. The PCR for the *M. ulcerans* IS*2404* region has a high sensitivity and specificity to detect Buruli ulcer (*35*). A study in Ghana showed that >50% of 2,203 clinically diagnosed Buruli ulcer cases were actually not Buruli ulcer, as shown by PCR, culture, and histology (*36*). To avoid overdiagnosis of Buruli ulcer and unnecessary preemptive antimicrobial therapy, we suggest performing PCR in all cases before the initiation of chemotherapy, which is not the current common practice in many countries because of unavailability of the assay and long turnaround time for results where it is available. A point-of-care diagnostic tool is needed and would greatly improve confirmation of Buruli ulcer cases in the field. Currently, simpler methods such as loop-mediated isothermal amplification assay and fluorescent thin layer chromatography are being tested in some treatment centers in Africa (*37*).

Recent advances in our understanding of *M. ulcerans* suggest that lesion size is not necessarily a predictor for delayed manifestation, as was previously thought. It is more of a predictor for treatment outcome, because it reflects disease severity and is associated with increased disabilities and difficulties in treatment (*32*,*38*). Furthermore, presence of an ulcerative lesion should not be interpreted as caused solely by late reporting. Buruli ulcer can manifest as a nodule, plaque, edematous lesion, or ulcer, and the factors that contribute to each occurrence are unclear; perhaps the route of transmission and specific host immune response are factors determining this. The ulcer is not necessarily a late stage of either of the other manifestations and can occur without an evident previous nodular stage.

Future programmatic targets should be implemented to assess progress on Buruli ulcer disease control. To address the challenges of Buruli ulcer, these targets should focus on secure diagnosis (PCR confirmation), early case finding (duration of disease reported by patients), case severity (category III lesions), effective treatment (application of oral antimicrobial regimens and 100% completion rate), and reduction of sequelae and disability (scarring, movement limitation). Strengthening active epidemiologic surveillance in underserved areas is as paramount as research into the ecology, transmission, and epidemiology of Buruli ulcer.

This study had several limitations. First, we analyzed only data officially reported to WHO. Buruli ulcer cases did occur in the 2010–2017 period in some other countries than those described in this study, as published literature suggests (*15*), but these cases might not have been reported to WHO for reasons such as local practices, weak health and surveillance systems, or neglect. All countries should be encouraged to report accurate data to WHO so that appropriate support in disease control can be provided. Low case numbers do not always indicate a low disease burden, as in the case of inadequate reporting of disease.

Integrated care for neglected tropical skin diseases is an increasingly popular approach recommended by WHO (*39*–*41*). We expect integrated case search for these diseases to improve early case detection of Buruli ulcer. An emphasis on precise reporting of cases, with a focus on disease-endemic regions and analysis and mapping of collected data, will ensure sound data for policy planning and Buruli ulcer disease control. As of 2019, countries have been enabled to directly enter Buruli ulcer epidemiologic information into DHIS2, facilitating easier reporting; we expect timeliness, completeness, and use of data to improve. Furthermore, information from the BU02 form is available for most cases from Buruli ulcer–endemic regions; this information, which provides insights into the subnational epidemiology of Buruli ulcer, can give a clearer picture of local epidemiology and would enable comparison of programmatic indicators across health districts or even single health facilities.

Because Buruli ulcer is an environmental disease following unknown ecologic trends, rapid case detection and good treatment are the mainstay components in reducing death and disability associated with the disease. In the framework of universal health coverage, each Buruli ulcer patient should have access to comprehensive treatment, including antimicrobial medication and wound care.
